# Comparing Functional Outcomes Following TORS With Free Flap Reconstruction Using a Radial Forearm vs. Anterolateral Thigh Flap

**DOI:** 10.1002/hed.70039

**Published:** 2025-09-12

**Authors:** Praneet Kaki, Doreen Lam, Neel R. Sangal, Ryan M. Carey, Karthik Rajasekaran, Ara Chalian, Robert M. Brody, Gregory S. Weinstein, Steven B. Cannady

**Affiliations:** ^1^ Sidney Kimmel Medical College Thomas Jefferson University Philadelphia Pennsylvania USA; ^2^ Department of Otolaryngology—Head and Neck Surgery Cleveland Clinic Cleveland Ohio USA; ^3^ Department of Otolaryngology—Head and Neck Surgery University of Pennsylvania Philadelphia Pennsylvania USA

**Keywords:** anterolateral thigh, dysphagia, FOIS, free flap, functional outcomes, radial forearm, TORS

## Abstract

**Background:**

Free flap reconstruction (FFR) following transoral robotic surgery (TORS) is commonly performed using radial forearm free flaps (RFFF). This study compares patterns of functional recovery between RFFFs and anterolateral thigh flaps (ALTFs).

**Methods:**

Retrospective cohort study of patients with oropharyngeal cancer who underwent TORS with FFR (2010–2023) at a tertiary care center. A 1:4 ALTF:RFFF propensity score match was performed. Functional Oral Intake Scale (FOIS) assessed outcomes.

**Results:**

The 105 patients were included. ALTF patients had lower FOIS at 6 months (3[2.0, 5.0] vs. 5.0[2.0, 6.0], *p* = 0.045) and 1 year (5.0[2.0, 6.0] vs. 6.0[5.0, 6.8], *p* = 0.045), with higher PEG tube rates. ALTFs were used for larger defects (62.95 ± 20.25 cm^2^ vs. 51.17 ± 15.11 cm^2^, *p* = 0.014). Donor site morbidity or postoperative complications were similar between cohorts.

**Conclusions:**

ALTFs are suitable for larger defects and lead to slower early functional recovery with higher PEG tube rates compared to RFFFs, with no difference in complications.

## 1 Introduction

Human papillomavirus (HPV) infection accounts for over 65% of oropharyngeal squamous cell carcinoma (OPSCCs) in North America, with an increasing incidence reflected by an age‐standardized incidence rate (ASIR) of 3.41 per 100 000 in males and 0.71 per 100 000 in females [1]. The advent of TORS in 2005 offered head and neck (H&N) surgeons a minimally invasive surgical technique for resecting tumors of the oropharynx and oral cavity while reducing complications and recovery time [2, 3, 4]. Resection of large, complex tumors in this region often results in corresponding defects that require soft tissue coverage with a local flap or free flap. Microvascular free flap reconstruction (FFR) is indicated for large oropharyngeal or oral cavity defects, extension into the soft palate, prior radiation to the H&N, and exposure of the retropharyngeal carotid artery.

With success rates of FFR in H&N reconstruction reaching 91%–99%, there is increasing focus on using FFR to optimize speech and swallowing functional recovery in patients [5, 6, 7]. Dysphagia affects up to 50% of H&N cancer survivors [8]. Quality of life (QoL) and overall functional status in these patients are significantly impacted by swallowing function, nutritional intake, and the incidence of common complications such as xerostomia, aspiration, and velopharyngeal insufficiency (VPI) [9, 10, 11, 12]. In a survey of H&N patients, percutaneous endoscopic gastrostomy (PEG) tube dependence was the strongest negative predictor of QoL [10]. Given the favorable oncologic outcomes in HPV‐positive H&Ns, there is increased emphasis on nutrition and swallowing function, making it essential to understand the impact of different interventions on long‐term functional outcomes.

FFR for oropharyngeal and oral cavity defects is most commonly performed using the radial forearm fasciocutaneous flap (RFFF). The RFFF provides a thin and pliable tissue that is advantageous for intraoral reconstruction. However, the harvest of the RFFF involves the sacrifice of a major vessel in the radial artery and possible severe donor site morbidity [13, 14]. The conspicuous donor‐site scar on the visible forearm donor site could pose cosmetic concerns and social stigma for some patients [15]. The anterolateral thigh flap (ALTF) is alternatively used at our institution that is based on the septocutaneous vessels or musculocutaneous perforators from the descending branch of the lateral circumflex femoral artery [16]. It is used for larger defects or if an RFFF is contraindicated for reasons including dorsal palmar arch insufficiency, need for radial artery preservation, previous surgery, or concern for donor‐site morbidity [17]. ALTFs offer certain advantages compared to the RFFF, such as low donor site morbidity and the ability to be thinned before or after ligation of the vascular pedicle [18, 19, 20]. However, considering the bulkier nature of the ALTF, there may be some concern for negative impacts on pharyngeal function following reconstruction compared to the RFFF.

Literature assessing functional outcomes following TORS with FFR is limited. A recent retrospective review of 241 patients performed at our institution demonstrated that FFR after TORS does not increase long‐term swallowing morbidity, as patients generally achieve near‐normal oral intake within 1 year [21]. A systematic review of 21 small‐cohort studies found that FFR following TORS achieves comparable functional outcomes as non‐TORS free‐flap reconstructions and TORS without free‐flap reconstructions [22]. Whether the choice of donor site for FFR within this patient population affects long‐term functional outcomes has yet to be studied. This study utilized the functional oral intake scale (FOIS), a validated clinical measure of swallow function and oral intake, to compare the functional outcomes in patients undergoing TORS with FFR with either RFFF or ALTFs.

## 2 Materials and Methods

This is a retrospective cohort study approved by the institutional review board at the University of Pennsylvania (IRB 826710).

### 2.1 Cohort Selection

Electronic medical records (EMR) of patients who underwent TORS with FFR at the University of Pennsylvania between December 2010 and January 2023 were reviewed. The 276 total patients were identified, of whom five who died within 30 days of surgery (three from myocardial infarction and two from pulmonary embolism) and four who received ulnar forearm free flaps were excluded. Patients were stratified by type of flap received: ALTF or RFFF. A 1:4 propensity score match (PSM) was performed to match the 21 patients who received ALTFs with those who received RFFFs based on demographics, tumor characteristics, and adjuvant/prior treatment (e.g., radiotherapy, chemoradiotherapy). A total of 105 patients were included in the final analysis following PSM. Flap choice was based on institutional protocols and patient‐specific factors, with RFFF preferred for smaller intraoral defects and ALTF selected for larger defects, need for radial artery preservation, inadequate palmar arch perfusion, or prior forearm surgery. Patients without at least one Speech‐Language Pathology (SLP) follow‐up visit were excluded.

### 2.2 Demographics and Clinicopathologic

Basic demographic data including age, sex, race, smoking history, and the Charlson comorbidity index (CCI) was reported. Clinicopathologic information included primary tumor site and pathologic tumor (pT) stage according to the American Joint Committee on Cancer (AJCC) 8th Edition classification. HPV‐related oropharyngeal squamous cell carcinomas (OPSCC) were classified according to p16 immunohistochemistry staining. National Comprehensive Cancer Network guidelines were followed to inform the administration of adjuvant treatment, according to the presence of concerning features, such as positive surgical margins, extent of nodal disease, and extranodal extension. Adjuvant therapy started 6 weeks after surgery and typically lasted 6–7weeks. FFR indication, flap surface area (cm^2^), flap takeback, and failures were reported.

### 2.3 Outcome Measures

**FIGURE 1:**
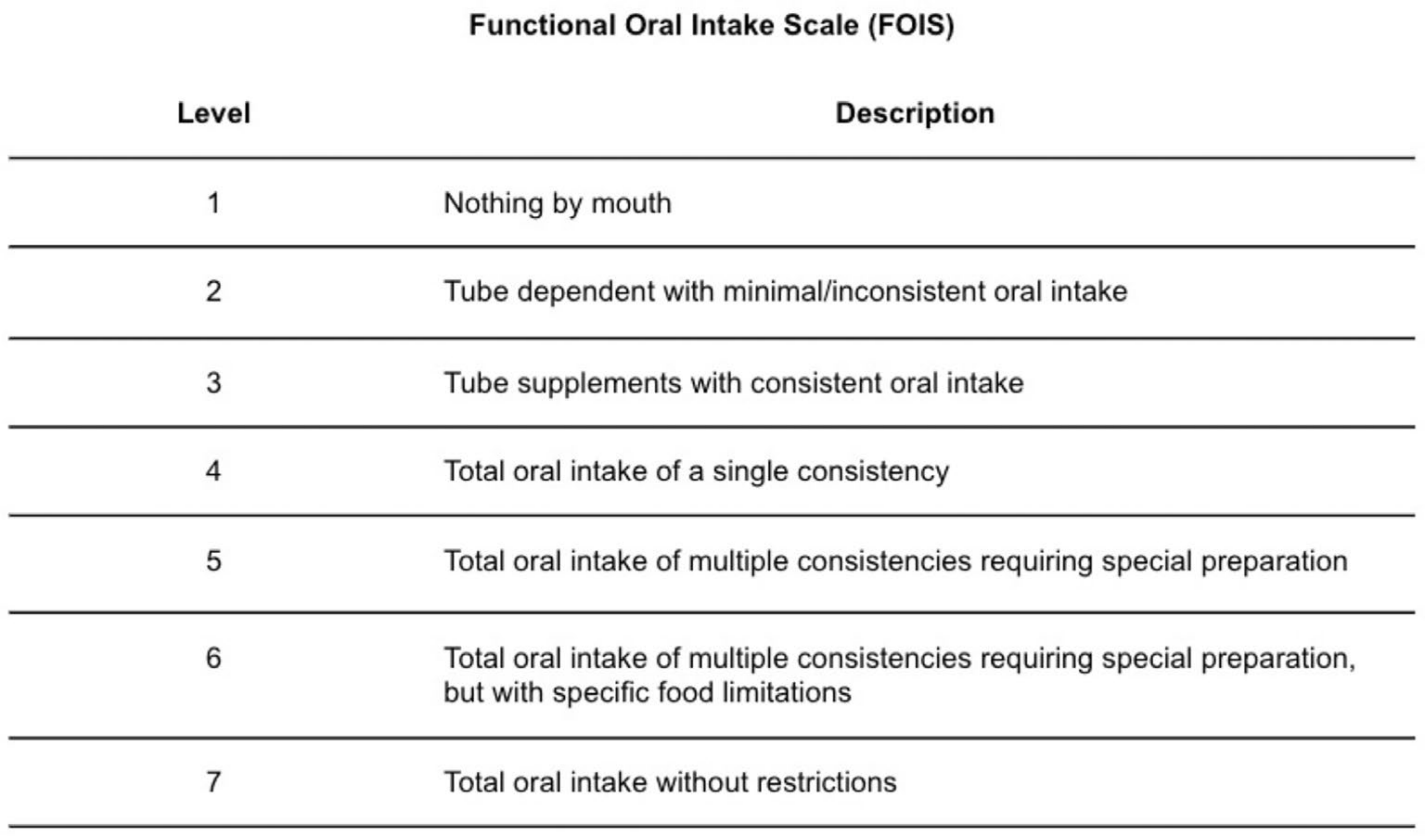
Functional oral intake scale (FOIS) levels. FOIS is a validated scale to assess nutritional intake in patients with notable dysphagia based on the ability to tolerate oral intake and various consistencies of food.

The FOIS was the primary outcome used to measure the ability to tolerate oral intake. This scale is scored from 1 to 7, with 1 representing no oral intake and 7 indicating full oral intake without restriction (Figure 1). Oral intake without the need for feeding tube supplementation is achieved at a FOIS of 4, and near‐normal oral intake is indicated by a FOIS of 6. FOIS was collected at baseline (closest to the time of surgery) and 2–3 weeks, 3–4 weeks, 6 weeks, 3 months, 6 months, and 1 year following surgery, based on our standard clinical follow‐up schedule and availability of data in the EMR. Information regarding dietary intake as documented in the EMR by SLPs, oncology nurses, or H&N surgeons was collected to determine FOIS. There is a lack of patient‐reported outcome (PRO) data on swallowing function, as surveys were not routinely administered at our institution. However, the FOIS has demonstrated congruency with other PRO measures in patients with HNC [23, 24].

### 2.4 Statistical Analysis

Descriptive statistics were utilized to characterize FFR donor site cohorts. Continuous variables were represented as either mean (standard deviation) or median (interquartile range) and assessed using one‐way ANOVA and post hoc tests. Chi‐square (*χ* ^2^) and Fisher's exact tests were used to analyze categorical variables, displayed as frequency (percentage). To account for the repeated measures of FOIS over time within patients, a linear mixed‐effects model was constructed with FOIS as the dependent variable, and Donor Site, Timepoint, and their interaction as fixed effects, with a random intercept for each patient. This approach modeled changes in FOIS longitudinally while adjusting for within‐patient correlations. Estimated marginal means were compared between flap types at each postoperative time point. Multivariable linear regression was performed to identify predictors of 3‐month and 1‐year FOIS scores. Overall survival at the time of most recent follow‐up was estimated using the Kaplan–Meier method and compared using the log‐rank test between donor site groups. All statistical analysis was conducted in R Studio Version 2023.03. A *p*‐value < 0.05 was considered to be significant.

## 3 Results

### 3.1 Demographics and Disease Characteristics

Initially, 267 patients undergoing FFR following TORS resection were included, of which 26 (9.7%) received ALTFs and 241 (90.3%) received RFFFs. There were no differences in demographic and baseline characteristics including age, race, sex, smoking history, CCI, p16 positivity, pathologic T stage, and treatment history.

After 1:4 PSM, 105 patients were included in the final analysis, of which 84 (80%) received an RFFF and 21 (20%) an ALTF. The mean age was 61.3 years, 86% of patients were male, 82% were White, and 57% had a smoking history of at least 10 pack‐years (Table 1). The mean CCI was 2.63. 71% of the tumors were p16‐positive, and 48% were either pathologic T3 or T4 stage. The majority (64%) of tumors were located in the palatine tonsils, and 35% were located at the tongue base. AdjCRT and adjRT were administered to 30 (29%) and 33 (31%) patients, respectively. 20 (19%) had undergone prior radiation to the head and neck, and 22 (21%) did not undergo prior or adjuvant radiotherapy. All patients required a tracheostomy. Upon PSM, there were no significant differences in any of these baseline characteristics between the RFFF and ALTF cohorts. ALTFs had, on average, a larger average surface area than RFFF (ALTF: 62.95 cm^2^ [SD = 20.25] vs. RFFF: 51.17 [15.11], *p* = 0.014).

**TABLE 1:** Demographics and disease characteristics.

Characteristic	*N*	Overall, *N* = 105^a^	ALTF, *N* = 21^*a*^	RFFF, *N* = 84^a^	*p* ^b^
Age at surgery (years)	105	61.26 (10.50)	62.67 (9.86)	60.90 (10.69)	0.7
Race	105				> 0.9
Non‐White		19 (18%)	4 (19%)	15 (18%)	
White		86 (82%)	17 (81%)	69 (82%)	
Male	105	90 (86%)	18 (86%)	72 (86%)	> 0.9
Smoker	105	60 (57%)	12 (57%)	48 (57%)	> 0.9
Charleson comorbidity index	105	2.63 (1.22)	2.67 (1.59)	2.62 (1.12)	0.8
p16+	105	75 (71%)	15 (71%)	60 (71%)	> 0.9
pT	105				> 0.9
pT1/2		55 (52%)	11 (52%)	44 (52%)	
pT3/4		50 (48%)	10 (48%)	40 (48%)	
Tumor site	105				0.6
BOT		37 (35%)	9 (43%)	28 (33%)	
Retromolar trigone		1 (1.0%)	0 (0%)	1 (1.2%)	
Tonsil		67 (64%)	12 (57%)	55 (65%)	
Treatment history	105				> 0.9
Chemoradiotherapy		30 (29%)	6 (29%)	24 (29%)	
No adjuvant therapy		22 (21%)	4 (19%)	18 (21%)	
Prior CRT/RT		20 (19%)	4 (19%)	16 (19%)	
Radiation only		33 (31%)	7 (33%)	26 (31%)	
Flap surface area (cm^b^)	101	53.62 (16.90)	62.95 (20.25)	51.17 (15.11)	0.014
Soft palate involvement	92	18 (20%)	1 (5.3%)	17 (23%)	0.11
Retropharyngeal carotid	92	27 (29%)	7 (37%)	20 (27%)	0.4
Large tumor	98	18 (18%)	3 (16%)	15 (19%)	> 0.9
Prior radiation exposure	92	18 (20%)	1 (5.3%)	17 (23%)	0.11

^a^ Mean (SD); *n* (%).

^b^ Wilcoxon rank sum test; Fisher's exact test; Pearson's *χ* ^2^ test.

### 3.2 Post‐Operative Nutritional Support and Recovery of Oral Intake

Following PSM, patients were cleared for first oral intake at a median of 17 days postoperatively (IQR: 15.5–24.5) (Table 2). 93 (89%) patients received a DHT intraoperatively. PEG placement occurred in 34 (33%) cases intraoperatively or after failure to tolerate DHT removal, and was more common among patients who received an ALTF (52% vs. 28%, *p* = 0.031) and those who received prior radiotherapy or chemoradiotherapy to the H&N region (prior CRT/RT: 55% vs. no prior CRT/RT: 27%, *p* = 0.018). At 1 year after surgery, 19% of patients remained PEG‐tube dependent, and did not reach statistical significance when compared between patients who underwent ALTFs and RFFFs, likely due to the small sample size (ALTF: 33% vs. RFFF: 15%, *p* = 0.095). Patients experienced an average weight loss of 5.26% from baseline in the year after surgery (ALTF: −6.05% vs. RFFF: −5.23%, *p* = 0.7). The average time to decannulation was 16 (15–27) days (ALTF: 20 vs. RFFF: 16 days, *p* = 0.3), and only one patient was decannulated after 6 months.

**TABLE 2:** FOIS and nutritional outcomes.

Characteristic	*N*	Overall, *N* = 105^a^	ALTF, *N* = 21^a^	RFFF, *N* = 84^a^	*p* ^b^
Pre‐op FOIS	71	7.00 (6.00, 7.00)	7.00 (5.00, 7.00)	7.00 (6.00, 7.00)	0.7
2–3 weeks FOIS	102	2.00 (1.00, 4.00)	2.00 (1.00, 2.00)	2.00 (1.00, 4.00)	0.2
3–4 weeks FOIS	86	4.00 (2.00, 5.00)	2.00 (2.00, 2.00)	4.00 (2.00, 5.00)	0.005
~6 weeks FOIS	85	4.00 (2.00, 5.00)	3.00 (2.00, 4.00)	5.00 (2.00, 5.00)	0.027
3 months FOIS	87	5.00 (2.00, 6.00)	3.00 (2.00, 4.50)	5.00 (2.00, 6.00)	0.022
6 months FOIS	88	5.00 (2.00, 6.00)	3.00 (2.00, 5.00)	5.00 (2.00, 6.00)	0.045
1 Year FOIS	75	6.00 (4.00, 6.00)	5.00 (2.00, 6.00)	6.00 (5.00, 6.75)	0.045
Near normal intake at 1 year (FOIS 6/7)	75	39 (52%)	6 (35%)	33 (57%)	0.12
Days to first oral intake	103	17.00 (15.50, 24.50)	21.00 (16.00, 24.00)	16.50 (15.00, 24.75)	0.2
Dobhoff tube placed	105	93 (89%)	18 (86%)	75 (89%)	0.7
PEG tube inserted	104	34 (33%)	11 (52%)	23 (28%)	0.031
PEG dependence at 1‐year	97	18 (19%)	6 (33%)	12 (15%)	0.095
PEG dependence at 2‐years	93	10 (11%)	2 (12%)	8 (11%)	> 0.9
% Weight change at 1 year from post‐op	77	−5.26 (−10.77, −1.03)	−6.05 (−10.34, 0.74)	−5.23 (−10.79, −1.73)	0.7
Time to decannulation	99	16.00 (15.00, 27.00)	20.00 (16.00, 25.25)	16.00 (15.00, 27.00)	0.3
Trach decannulation > 6 months	99	1 (1.0%)	0 (0%)	1 (1.2%)	> 0.9

^a^ Median (IQR); *n* (%).

^b^ Wilcoxon rank sum test; Pearson's *χ* ^2^ test; Fisher's exact test.

**FIGURE 2:**
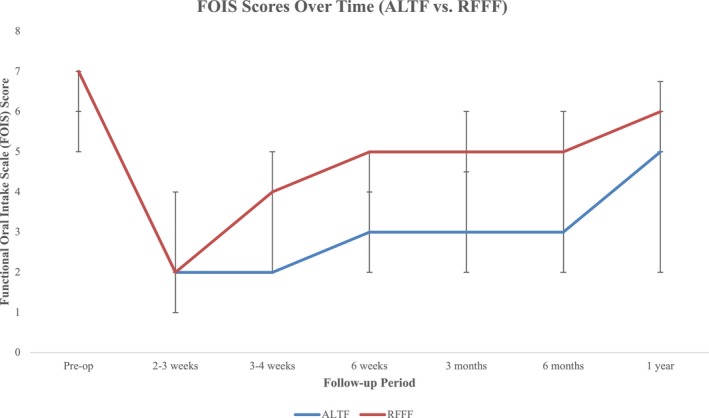
Functional oral intake scale (FOIS) scores over time by FFR donor site. [Color figure can be viewed at wileyonlinelibrary.com]

The median FOIS at baseline before surgery was 7.0 (IQR = 6.0, 7.0), from which patients demonstrated a decline to 2.0 (1.0, 4.0) at their follow‐up 2 to 3weeks after surgery (Table 2). Until this time point, there were no differences between the ALTF and RFFF. However, RFFF had higher FOIS scores than ALTF patients (RFFF: 4.0 [2.0, 5.0] vs. ALTF: 2.0[2.0, 2.0], *p* < 0.01) 3 weeks post‐operatively (Figure 2). While both groups progressively improved in their ability to tolerate oral intake, with increased FOIS at 6 months (5.0[2.0, 6.0]vs. 3[2.0, 5.0], *p* = 0.045) and 1 year (6.0[5.0, 6.8] vs. 5.0[2.0, 6.0], *p* = 0.045), RFFF patients experienced better swallowing outcomes throughout this period. To validate these trends while accounting for repeated measures, a linear mixed‐effects model was used to assess FOIS trajectories over time. This analysis confirmed that RFFF patients demonstrated significantly better recovery at 3–4 weeks (mean FOIS: 3.47 vs. 2.27, *p* = 0.018), 3 months (4.44 vs. 3.16, *p* = 0.0075), and 6 months (4.66 vs. 3.62, *p* = 0.035) compared to ALTF patients, although differences at 1 year (5.10 vs. 4.14, *p* = 0.062) were not statistically significant.

On multivariable linear regression, RFFF was independently associated with improved FOIS at 3 months (*β* = 1.4, 95% CI 0.7–2.2, *p* < 0.001) and 1 year (*β* = 0.99, 95% CI 0.2–1.8, *p* = 0.015), respectively, after adjusting for age, smoking status, tumor subsite, adjuvant treatment, and pathologic T stage (Table 3). At 1 year, near‐normal oral intake (FOIS ≥ 6) was tolerated in 57% of patients who received an RFFF and 35% of patients who received an ALTF (*p* = 0.12). 71% (75/105) of patients had 1‐year follow‐up data for FOIS available.

**TABLE 3:** Univariable and multivariable linear regression identifying predictors of 3‐month and 1‐Year FOIS.

3 month FOIS						
Covariate	Univariable			Multivariable		
	*β*	95% CI^a^	*p*	*β*	95% CI^a^	*p*
Age	−0.05	−0.10, −0.01	**0.011**	−0.02	−0.05, 0.01	0.2
Smoker	−0.06	−0.88, 0.76	0.9	—	—	—
BOT tumor site	−1.4	−2.2, −0.56	**0.001**	−0.61	−1.3, 0.05	0.071
RFFF	1.2	0.21, 2.1	**0.017**	1.4	0.70, 2.2	**< 0.001**
Treatment history						
No adjuvant therapy	—	—	—	—	—	—
Prior CRT/RT	−3.7	−4.9, −2.5	**< 0.001**	−3.6	−4.7, −2.5	**< 0.001**
Radiation only	−1.1	−2.1, 0.02	**0.046**	−1.1	−2.1, −0.19	**0.020**
Chemoradiotherapy	−2.0	−3.1, −0.93	**< 0.001**	−2.0	−3.0, −1.0	**< 0.001**
pT3/4	−0.78	−1.6, 0.02	0.056	—	—	—

1 year FOIS						
Covariate	Univariable			Multivariable		
	*β*	95% CI^a^	*p*	*β*	95% CI^a^	*p*
Age	−0.03	−0.07, 0.01	0.2	—	—	—
Smoker	−0.07	−0.95, 0.81	0.9	—	—	—
BOT tumor site	−0.66	−1.5, 0.22	0.14	—	—	—
RFFF	1.0	0.0–2.0	0.051	0.99	0.20, 1.8	**0.015**
Treatment history						
No adjuvant therapy	—	—	—	—	—	—
Prior CRT/RT	−3.5	−4.6, −2.3	**< 0.001**	−3.4	−4.5, −2.4	**< 0.001**
Radiation only	−1.3	−2.2, −0.31	**0.010**	−1.2	−2.1, −0.30	**0.010**
Chemoradiotherapy	−2.5	−3.6, −1.5	**< 0.001**	−2.5	−3.5, −1.5	**< 0.001**
pT3/4	0.08	−0.79, 0.95	0.9	—	—	—

*Note:* Bolded numbers are statistically significant *p* values.

^a^ CI, confidence interval.

### 3.3 Postsurgical Outcomes and Survival

Following PSM, rates of flap failure (9.5% vs. 1.2%, *p* = 0.10), flap takeback to the OR (19% vs. 14%, *p* = 0.7), trismus (15% vs. 18%, *p* > 0.9), xerostomia (40% vs. 39%, *p* > 0.9), velopharyngeal insufficiency (9.5% vs. 12%, *p* > 0.9), and aspiration confirmed by swallow study (14% vs. 18%, *p* > 0.9) were similar between patients who received an ALTF and those who received an RFFF (Table 4). There were no occurrences of pneumonia in association with aspiration.

**TABLE 4:** Postsurgical complications.

Characteristic	*N*	Overall, *N* = 105^a^	ALTF, *N* = 21^a^	RFFF, *N* = 84^a^	*p* ^b^
Free flap failure	105	3 (2.9%)	2 (9.5%)	1 (1.2%)	0.10
Flap takeback to OR	105	16 (15%)	4 (19%)	12 (14%)	0.7
Trismus	103	18 (17%)	3 (15%)	15 (18%)	> 0.9
Xerostomia	103	40 (39%)	8 (40%)	32 (39%)	> 0.9
Velopharyngeal insufficiency	105	12 (11%)	2 (9.5%)	10 (12%)	> 0.9
Aspiration confirmed by MBS/FEES	105	18 (17%)	3 (14%)	15 (18%)	> 0.9

^a^ *n* (%).

^b^ Fisher's exact test; Pearson's *χ* ^2^ test.

Flap donor site complications occurred in 26% of all patients following PSM (Table 5). Patients who received RFFFs had higher rates of experiencing any donor site complication compared to those who received ALTFs, although this difference did not reach statistical significance (29% vs. 14%, *p* = 0.2). Hypertrophic scarring, tendon exposure, functional impairment, and hyperpigmentation were noted in 2 (2.4%), 5 (6.0%), 7 (8.3%), and 1 (1.2%) patients who received RFFFs, respectively. Hematomas at the donor site were observed in 2 patients who received RFFFs and 1 patient who received an ALTF. Paresthesia/numbness at the flap donor site occurred in 13 (15%) patients who received RFFFs and 2 (9.5%) patients who received ALTFs (*p* = 0.7). There were no mortalities within 30 days following surgery. Kaplan Meier analysis demonstrated no difference in overall survival between patients receiving ALTFs and RFFFs at the time of most recent follow up (*p* = 0.11).

**TABLE 5:** Donor site morbidity.

Characteristic	*N*	Overall, *N* = 105^a^	ALTF, *N* = 21^a^	RFFF, *N* = 84^a^	*p* ^b^
Hypertrophic scarring	105	2 (1.9%)	0 (0%)	2 (2.4%)	> 0.9
Tendon exposure	105	5 (4.8%)	0 (0%)	5 (6.0%)	0.6
Hematoma	105	3 (2.9%)	1 (4.8%)	2 (2.4%)	0.5
Paresthesia/numbness	105	15 (14%)	2 (9.5%)	13 (15%)	0.7
Functional impairment	105	7 (6.7%)	0 (0%)	7 (8.3%)	0.3
Hyperpigmentation	105	1 (1.0%)	0 (0%)	1 (1.2%)	> 0.9
Any donor site complication	105	27 (26%)	3 (14%)	24 (29%)	0.2

^a^ *n* (%).

^b^ Fisher's exact test; Pearson's *χ* ^2^ test.

## 4 Discussion

FFR following resection of oropharyngeal and oral cavity cancers via TORS is an increasingly utilized technique for soft tissue reconstruction. Vast improvements in this procedure and the viability of free flaps have shifted focus onto functional outcomes and QoL in patients. However, FFR is a relatively uncommon approach for patients, typically reserved for patients with large complex oropharyngeal tumors, and studies assessing the impact of flap choice in this patient population are limited. In this propensity score‐matched analysis of 105 patients, those who received ALTFs experienced a relatively slower recovery in nutritional intake per FOIS and were more likely to receive a PEG tube following TORS. However, differences in long‐term swallowing function and PEG tube dependence did not reach statistical significance.

The RFFF is traditionally favored for FFR of the H&N due to its pliability, reliable anatomy, ease of harvest, and long pedicle length [25]. However, RFFF harvest raises concerns due to radial artery sacrifice and significant functional and cosmetic donor site morbidity [26]. Thus, the ALTF is a viable alternative that offers pliable tissue with adjustable thickness, availability of muscle, and less donor‐site morbidity [26, 27]. Indications for FFR via ALTF in our study population included prior RFFF morbidity, dorsal palmar arch insufficiency, need for radial artery preservation, and previous surgery. There are, however, other factors that must be considered during the decision‐making process for flap selection. Harvest of the ALTF requires substantial surgical experience and presents a relatively low, but non‐zero rate of non‐usability [28]. Moreover, ALTFs are difficult to harvest in patients with a high volume of subcutaneous tissue in the thigh, thereby restricting its use in obese individuals [25].

Our results demonstrated that patients receiving ALTFs experienced a slower recovery in swallowing function, tending to tolerate consistent oral intake but remaining tube feed dependent at 6 months (median FOIS 3.00). This trend was further confirmed by a linear mixed‐effects model, which demonstrated that RFFF patients experienced significantly better recovery in FOIS scores at 3–4 weeks, 3 months, and 6 months postoperatively compared to ALTF patients, although differences at 1 year were not statistically significant. Prior studies found that reconstruction of tongue defects with ALTF adversely impacted speech, swallowing, and chewing function and proposed that the bulky nature of this flap may hinder swallowing function due to reduced pliability and tongue‐to‐palate contact, potentially requiring a lengthy recovery [29, 30, 31, 32]. In our cohort, ALTFs, on average, had a larger surface area compared to RFFFs, which could contribute to the aforementioned hindrance of early swallowing function due to the excess bulk.

Further analysis at the 1‐year post‐operative timepoint continued to demonstrate a difference in swallowing function per the FOIS scale between patients receiving RFFFs and ALTFs. Albeit statistically significant, we consider the 1‐point difference in a FOIS of 5, indicating “Total oral intake of multiple consistencies requiring special preparation”, and a FOIS of 6, indicating “Total oral intake with multiple consistencies without special preparation, but with specific food limitations”, to be clinically insignificant and possibly limited by the small size of our ALTF cohort. Moreover, the higher rates of PEG tube placement in ALTF patients did not translate into an increased likelihood of long‐term PEG tube dependence, suggesting significant improvement in nutritional status. Similarly, a meta‐analysis by Ranganath et al. comparing RFFFs and ALTFs in oral cavity reconstruction reported no differences in speech, diet, and QoL, although reconstruction with RFFFs presented greater donor site complications [22]. Another retrospective cohort study by Zhao et al. of 80 patients undergoing FFR for oral and maxillofacial defects found that utilization of ALTFs achieved similar therapeutic effects, food intake, and University of Washington Quality of Life (UW‐QOL) to RFFF while reducing the incidence of postoperative complications such as temporary dysfunction, hyperplastic scar, permanent dysfunction, pigmentation, and pruritus [33].

Our study found no statistical difference in postsurgical and functional oral complications between patients who received RFFFs and ALTFs, consistent with existing literature [34, 35]. Although there was no statistically significant difference in donor site morbidity within our cohort, this could be due to our small cohort of patients who received ALTFs. Of note, none of the patients who received ALTFs in our cohort experienced hypertrophic scarring, tendon exposure, functional impairment, or hyperpigmentation. Previous studies suggest that FFR with RFFFs presents a greater risk of severe donor site morbidity, notably scarring and functional impairment [13, 14]. These findings suggest that the ALTF is a viable option for reconstruction following TORS since it has flap failure and complication rates similar to those of the RFFF. Moreover, while not directly within the scope of this study, we acknowledge the utility of the oropharyngeal defect classification proposed by De Almeida et al., in which most defects in our cohort correspond to stage III or IV [36]. Future efforts to develop more granular classification systems for extensive oropharyngeal defects may enhance reconstructive planning and facilitate cross‐study comparisons.

### 4.1 Strengths

This is the largest cohort study to date comparing functional outcomes between patients who underwent TORS with FFR using either an RFFFF or ALTF. The high volume of OPSCC patients at our institution allowed for a PSM to control for demographic and clinicopathologic factors that can influence functional outcomes. We were able to assess the progression of recovery in oral intake as measured by the FOIS at several time points throughout the year following surgery. Moreover, the FOIS itself offers additional reliability compared to subjective PROs as an objective measure of oral intake that characterizes nuanced aspects of swallowing physiology based on clinician assessment. Our study also broadens the scope of existing literature on TORS which is generally limited to early‐stage pT1 to pT2 oropharyngeal tumors by including pT3 to pT4 tumors, which constituted 48% of our study population. Surgeons at our institution deemed these advanced tumors to be amenable to surgical resection and microvascular reconstruction without compromising functional outcomes.

### 4.2 Limitations

Although a PSM analysis was conducted to analyze results, the retrospective observational design of our study nevertheless raises the possibility of unknown confounding factors and bias. Our sample size is relatively small (*N* = 105), reflecting the limited number of patients who underwent FFR using an ALTF. Moreover, ulnar forearm flaps, which may offer relatively less donor site morbidity and warrant further study, were excluded due to limited numbers (*N* = 4) in our institutional cohort during the study timeframe. Additionally, they were not combined with RFFFs into a single forearm flap category, given significant anatomical differences, including reduced tissue bulk and surface area, as well as a shorter vascular pedicle relative to RFFFs. Furthermore, although pT3–4 stage and the need for bilateral neck dissection have traditionally been considered contraindications to TORS, our academic center offers surgically intensive approaches for select patients. While this may limit generalizability, we believe that the inclusion of these patients illustrates the utility of TORS with FFR in advanced oropharyngeal malignancies.

Limitations inherent to the FOIS scale include its lack of consideration for weight changes and patient‐reported assessments of swallowing ability and nutritional satisfaction, which contribute to overall QoL. Additionally, while the FOIS scale captures functional oral intake, it does not directly measure objective swallow physiology, and our study did not collect instrumental swallowing assessments such as videofluoroscopic swallow studies or fiberoptic endoscopic evaluations of swallowing, which may have provided additional insight into swallowing function. Nevertheless, our choice to characterize outcomes by the FOIS was predicated on its apparent consistency with patient‐reported measures of functional nutritional intake. Overall and disease‐free survival outcomes were not measured in our study. It is important to understand functional ability in the context of oncological outcomes to better inform treatment planning and patient decision‐making.

## 5 Conclusions

TORS followed by FFR is a reliable therapeutic modality to manage OPSCCs without compromising functional outcomes. FFR using ALTFs leads to a relatively slower nutritional intake recovery with higher rates of PEG insertion with no increase in major functional complications. However, we deem the difference in nutritional tolerance at 1 year to be clinically insignificant. Thus, ALTFs are a reasonable choice for reconstruction in oropharyngeal cancer patients with contraindications to RFFF or larger defects requiring additional tissue bulk.

## Author Contributions

**Praneet Kaki:** data analysis and interpretation (lead), design of data (equal), methodology (equal), writing – original draft (equal), final approval of version published (equal); **Doreen Lam:** data analysis and interpretation (lead), design of data (equal), methodology (equal), writing – original draft (equal), final approval of version published (equal); **Neel R. Sangal:** methodology (lead), writing – review and editing (equal), final approval of draft (equal); **Ryan M. Carey:** methodology (equal), final approval of draft (equal); **Karthik Rajasekaran:** methodology (equal), final approval of draft (equal); **Ara Chalian:** methodology (equal), final approval of draft (equal); **Robert M. Brody:** methodology (equal), final approval of draft (equal); **Gregory S. Weinstein:** methodology (equal), final approval of draft (equal); **Steven B. Cannady:** conceptualization (lead), methodology (equal), writing – review and editing (equal), final approval of draft (equal).

## Conflicts of Interest

The authors declare no conflicts of interest.

## Data Availability

The data that support the findings of this study are available on request from the corresponding author. The data are not publicly available due to privacy or ethical restrictions.
